# Fièvre bilieuse hémoglobinurique (FBH) de révélation tardive: à propos d'un cas au CHU de Dakar

**DOI:** 10.11604/pamj.2015.22.301.8176

**Published:** 2015-11-25

**Authors:** Aliou Thiongane, Aliou Abdoulaye Ndongo, Papa Moctar Faye, Assane Sylla, Younoussa Kéïta, Djibril Boiro, Idrissa Basse, Ndèye Ramatoulaye Diagne Guèye, Ousmane Ndiaye

**Affiliations:** 1 Hôpital d'Enfants Albert Royer, Dakar, Sénégal; 2 Hôpital d'Enfants de Diamniadio, Dakar, Sénégal; 3 Service de Pédiatrie de l'Hôpital Aristide Le Dantec, Dakar, Sénégal; 4 Service de Pédiatrie de l'Hôpital Abass Ndao, Dakar, Sénégal

**Keywords:** Paludisme grave, hémoglobinurie, quinine, severe malaria, hemoglobinuria, quinine

## Abstract

La fièvre bilieuse hémoglobinurique (FBH) est une forme grave du paludisme; caractérisée par la survenue d'une hémolyse intravasculaire aiguë se traduisant par une anémie hémolytique, une insuffisance rénale aiguë et une hypovolémie. Son diagnostic repose essentiellement sur la clinique notamment la couleur des urines d'aspect rouge porto. Nous rapportons un cas de fièvre bilieuse hémoglobinurique chez un jeune garçon de 10 ans originaire et vivant dans une zone d'endémie palustre, colligé dans un CHU de Dakar (Sénégal). V.G est un garçon de 10 ans qui était hospitalisé pendant 2 jours au service des urgences dans un centre de santé de proximité pour une fièvre associée à des vomissements qui évoluaient depuis 3 jours. Le test de diagnostic rapide (TDR) au paludisme était positif à la présence d'antigènes de Plasmodium falciparum dans le sang. Un traitement à base de quinine injectable (25 mg/kg/j), a été entrepris pendant 03 jours. Au quatrième jour, sont apparues une hémolyse intravasculaire aiguë et une hémoglobinurie avec une insuffisance rénale aiguë anurique. La goutte épaisse était revenue négative et le traitement par la quinine injectable arrêté. Le relais a été fait avec les dérivés de l'artémisinine. Trois (03) séances d'hémodialyse ont été réalisées. L’évolution était favorable, par la reprise de la diurèse et la normalisation de la fonction rénale. La FBH existe encore dans notre pratique quotidienne. Il faut y penser devant l'apparition brutale d'une hémolyse avec insuffisance rénale aiguë et urines rouges porto au cours du traitement d'un paludisme.

## Introduction

La fièvre bilieuse hémoglobinurique (FBH) est une forme grave du paludisme, rare chez l'enfant [1]. Elle est caractérisée par la survenue d'une hémolyse intravasculaire aiguë se traduisant cliniquement par une anémie hémolytique, une insuffisance rénale aiguë et une hypovolémie. Son diagnostic repose essentiellement sur la clinique notamment la couleur des urines d'aspect rouge porto. La fièvre bilieuse hémoglobinurique est une complication du paludisme grave à Plasmodium falciparum qui serait déclenchée par la prise d'antipaludique. Elle a été décrite aux trois aminoalcools tels que la quinine, l'halofantrine et la mefloquine [2]. C'est une pathologie rare dont l'incidence peut atteindre 7,13‰ populations dans certaines zones d'Afrique [2]. Sa morbi-mortalité est élevée, en rapport avec la survenue brutale d'une insuffisance rénale, d'une anémie hémolytique et d'une hypovolémie conduisant au décès dans 23 à 26% des cas [2, 3]. Elle a été décrite pour la première fois chez les sujets européens expatriés non immuns vivant en zones d'endémie palustre [4–6]. Rare chez les indigènes, elle a été décrite au Sénégal et en Asie de l'ouest [7]. Nous rapportons un cas de fièvre bilieuse hémoglobinurique chez un garçon de 10 ans originaire et vivant en zone d'endémie palustre.

## Patient et observation

V.G est un garçon de 10 ans né et vivant en zone tropicale d'endémie palustre. Il a été hospitalisé pendant 2 jours au service des urgences dans un centre de santé de proximité pour une fièvre associée à des vomissements qui évoluaient depuis 3 jours. Il avait bénéfice d'un test de diagnostic rapide (TDR) du paludisme qui était positif traduisant la présence d'antigène de Plasmodium falciparum dans le sang. Ceci avait motivé la mise en route d'un traitement antipalustre à base de quinine injectable à raison de 25 mg/kg/jour. Devant l'aggravation clinique marquée par la survenue d'une altération de la conscience au 3 ^ème^ jour, il a été référé dans notre structure sanitaire où il a été hospitalisé au service des urgences. A l'arrivée l'examen clinique mettait en évidence une fièvre à 39°C, un trouble de la conscience avec un Glasgow à 11/15, une pâleur des muqueuses, un subictère conjonctival, il n'y avait pas œdème des membres inférieurs. Les urines étaient concentrées avec une diurèse à 1,2 ml/kg/h, les selles normales. Le poids était de 27 kg, la fréquence respiratoire à 40 c.p.m avec des râles bronchiques, la fréquence cardiaque à 120 b.p.m, la TA à 110/70 mm Hg. Le TDR du paludisme restait positif, la goutte épaisse était négative. Ceci avait motivé une admission aux soins intensifs pédiatriques où le traitement antipalustre a été poursuivi avec de la quinine à la même dose et de l'oxygène en discontinu. L’évolution a été marquée par la survenue brutale, au 2 ^ème^ jour d'hospitalisation correspondant au quatrième jour après le début de la maladie, d'un ictère franc, d'une accentuation de la pâleur des muqueuses et d'urines foncées (couleur acajou) avec une anurie à 0,46 ml/kg/h. La conscience était légèrement perturbée, l’état hémodynamique stable avec une TA à 120/80 mm Hg. L'examen des urines à la bandelette avait mis en évidence une hémoglobinurie à 2 croix, bilirubinurie 3 croix, protéinurie 2 croix, une absence d'urobilinogènes, d'hématies et de leucocytes, selles normales. Les examens biologiques mettaient en évidence une hyperleucocytose à 20300 éléments/mm ^3^, une anémie normochrome normocytaire avec un taux d'hémoglobine à 7,2 g/dl (Hb de départ), un taux de plaquettes normal à 194 000/mm ^3^. Au frottis sanguin on retrouvait une absence de schizocytes, le test de Coombs direct était négatif. Test d'Emmel négatif, urée: 1,27 g/l, créatininémie: 46 mg/l, protéinurie: 21,3 mg/kg/24h, ASAT: 334 UI/l, ALAT: 185 UI/l, CRP à 92 mg/L, ionogramme sanguin: 128 mEq/l de sodium et 5 mEq/l de potassium, TP: 100%, INR: 0,99, TCA: 29,5, TQ: 12 sec. Taux de bilirubine sanguine à 200 mg/l à prédominance non conjuguée. L’échographie abdominale avait montré: discrète dédifférenciation rénale, épanchement pleural bilatéral et péritonéal, œdème pariétal et boue vésiculaire. La biopsie rénale avait montré une nécrose tubulaire aiguë discréte à modérée. Ce tableau avait nécessité la réalisation de 3 séances d'hémodialyse avec transfusion de concentrés érythrocytaires. Le traitement antipalustre a été relayé par un dérivé de l'artémisinine par voie intramusculaire. L’état clinique et la fonction rénale s’étaient normalisés au bout de 5 jours.

## Discussion

La fièvre bilieuse hémoglobinurique (FBH) est une hémolyse aiguë intravasculaire survenant lors du traitement de l'accès palustre à Plasmodium falciparum [2]. Sa physiopathologie est complexe et n'est pas encore bien connue, mais selon certains auteurs la maladie serait secondaire à une double sensibilité des hématies à la quinine et au plasmodium falciparum ce qui provoquerait l'hémolyse aiguë [8]. D'autres facteurs sont associés à la survenue de la FBH notamment une immunité anti-malaria inadéquate, déficit en G6PD et un mauvais usage de la quinine [4]. Les facteurs de risque majeurs sont la quinine, une parasitémie basse et la saison des pluies [9]. Elle survient habituellement chez les sujets originaires de zones non impaludées durant leur séjour en zone d'endémie palustre [6]. Le tableau clinique est stéréotypé, survenant durant les 24 à 48 heures après l'administration de médicaments antipaludéens tels que la quinine, l'halofantrine, la mefloquine [10]. Elle se manifeste par la survenue brutale d'une émission d'urines rouge porto, un ictère, une pâleur, des nausées et une insuffisance rénale aiguë. L'anémie est d'emblée sévère. D'autres symptômes peuvent être observés tels que des douleurs abdominales, des vomissements, une hépatosplénomégalie, une dyspnée, une tachycardie, un malaise généralisé et des vertiges. Le tableau clinique peut aussi être asymptomatique [11, 12]. L'atteinte rénale serait secondaire à une nécrose tubulaire aiguë. La parasitémie est absente ou faible [8]. La coloration rouge porto des urines est le signe le plus constant. Bodi, en République Démocratique du Congo (RDC) retrouvait cet aspect des urines chez tous les patients atteints [9]. Notre observation se singularise par la survenue de la FBH chez un patient habitant en zone d'endémie palustre et un délai d'apparition des signes cliniques tardif, 72 heures après l'administration de quinine. En RDC, Bodi a rapporté dans une série de 43 cas de FBH que la majeure partie, 95%, sont secondaires à la prise de quinine [9]. L'insuffisance rénale aiguë est secondaire à une nécrose tubulaire objectivée par l'examen anatomo-pathologique du prélèvement de la ponction biopsie rénale. L'anémie était sévère à 7g/dl ayant nécessité une transfusion sanguine. La sévérité de l'anémie et l'insuffisance rénale augmentent le risque de survenue d'une FBH [9]. Il n'y avait pas de thrombopénie, l'examen du frottis sanguin était normal et le test de Coombs direct négatif ce qui nous a permis d’éliminer une hémolyse d'origine auto-immune et un syndrome hémolytique urémique. La densité parasitaire était indétectable avec une goutte épaisse négative, cependant la recherche d'antigènes dans le sang par le test rapide de diagnostic du paludisme était positive. L'hyperleucocytose observée, la CRP positive et les râles bronchiques pourraient traduire une surinfection bactérienne pulmonaire associée et avait nécessité la mise sous antibiothérapie empirique avec de l'amoxicilline par voie intraveineuse. Le traitement a consisté à un arrêt immédiat du traitement par la quinine et un relais par un dérivé de l'artémisinine par voie intramusculaire. Cette molécule a été bien tolérée chez notre patient contrairement à ce qui a été rapporté dans la littérature. Certains cas de FBH seraient secondaires aux dérivés de l'artémisinine [13]. La prise en charge de l'insuffisance rénale consistait en une restriction hydrique aux besoins de maintenance. Une hémodialyse a été réalisée. Bodi [9], dans sa série, a eu recours à la dialyse péritonéale chez 2 patients. L’évolution était favorable avec reprise de la diurèse, normalisation de la fonction rénale, et correction de l'anémie.

## Conclusion

La FBH existe encore dans notre pratique quotidienne. Elle est secondaire à l'utilisation de quinine dans le traitement du paludisme à plasmodium falciparum. Il faut y penser devant l'apparition brutale d'une hémolyse avec insuffisance rénale aiguë et urines rouges porto au cours du traitement d'un paludisme.
